# Reevaluating the Ethical Issues in Porcine‐to‐Human Heart Xenotransplantation

**DOI:** 10.1002/hast.1419

**Published:** 2022-10-13

**Authors:** Henry Silverman, Patrick N. Odonkor

**Keywords:** xenotransplantation, ethics, selection criteria, informed consent, psychosocial criteria, risks and benefits, bioethics, pig‐to‐human heart transplant

## Abstract

*A major limiting factor with heart allotransplantation remains the availability of organs from deceased donors. Porcine heart xenotransplantation could serve as an alternative source of organs for patients with terminal heart failure. A first‐in‐human porcine xenotransplantation that occurred in January 2022 at the University of Maryland Medical Center provided an opportunity to examine several ethical issues to guide selection criteria for future xenotransplantation clinical trials. In this article, the authors, who are clinicians at UMMC, discuss the appropriate balancing of risks and benefits and the significance, if any, of clinical equipoise. The authors also review the alleged role of the psychosocial evaluation in identifying patients at an elevated risk of posttransplant noncompliance, and they consider how the evaluation's implementation might enhance inequities among diverse populations. The authors argue that, based on the principle of reciprocity, psychosocial criteria should be used, not to exclude patients, but instead to identify patients who need additional support. Finally, the authors discuss the requirements for and the proper assessment of informed and voluntary consent from patients being considered for xenotransplantation*.

## Article

Cardiac transplantation is the preferred treatment option for patients with advanced heart failure.^1^ However, the limited supply of organs from deceased human donors combined with the growing number of patients seeking organs mean that many potential recipients are deprived of this option. Despite efforts to close this gap, such as by broadening the criteria for deceased donors, the shortage of organs remains unresolved. A left ventricular assist device (LVAD) can serve as a bridge to transplantation and, more recently, thanks to technological advances, can sometimes offer a long‐term alternative to a transplant, but many patients do not qualify for an LVAD due to structural and functional reasons, and the quality‐of‐life improvements possible with an LVAD are in any event questionable.^2^ Consequently, an alternative source of organs is needed for patients suffering from terminal organ failure.^3^

Recent advances in porcine heart xenotransplantation (transplantation of a pig's heart into a human recipient) could address this need. Advancements in genetic engineering of donor animals’ genomes, coupled with improvements in immunological treatments, have made it possible to prevent immune rejection, which has been the chief obstacle to xenotransplantation.

Given these accomplishments, along with recent successes in porcine xenograft transplantation with nonhuman primates, the U.S. Food and Drug Administration (FDA) granted emergency authorization to the University of Maryland Medical Center on December 31, 2021, for a first‐in‐human porcine heart xenotransplantation, which was performed on January 7, 2022. The patient, who was not eligible for a heart allotransplant (human‐to‐human transplantation) due to a history of noncompliance with a recommended medical regimen and was unsuitable for an LVAD because of arrhythmias, survived for over two months before succumbing to heart failure. The experience showed the promise of xenotransplantation. It also attested to the risks. For example, although the exact cause of the patient's death requires further investigation, tests performed shortly after the xenotransplant procedure revealed a latent porcine cytomegalovirus that had infected the pig and might have been responsible for the patient's heart dysfunction. The viral infection occurred even though the pig had been bred in a virus‐free environment and the viral testing performed on the pig prior to the transplant was negative. Alternatively, an untoward reaction to an antirejection drug might have contributed to the patient's heart failure.^4^

Even with this uncertainty about why the patient died, there will be ongoing attempts at xenotransplantation. This prospect, in conjunction with additional animal experiments as mandated by the FDA, suggests that the time has come to discuss the design of the forthcoming clinical xenotransplantation trials.^5^

Such clinical trials would require continued refinement of a regulatory framework for interspecies transplantation. Such a framework, which the FDA has begun to outline, would attempt to ensure the efficacy and safety of xenotransplantation products and procedures and to provide strategies to measure their identity, purity, and potency; develop methods to assess infectious disease risks associated with xenotransplantation products and porcine donor animals; review current strategies to control xenotransplant rejection; and address the appropriate surveillance monitoring techniques of potential viral transmissions posttransplant to ensure patient and societal safety from zoonotic infections.^6^

In addition to a regulatory framework, ethical guidelines are needed to answer the question, when is it ethically permissible to involve patients in xenotransplantation, specifically, in the context of clinical trials?

In this article, we approach this question informed by the experiences of the transplant team and other medical specialists confronted with the unique issues surrounding the selection of the pig‐human xenotransplant patient at the University of Maryland Medical Center, where one of us, Henry Silverman, is a physician and ethicist who chairs the clinical ethics committee and the other, Patrick Odonkor, is a cardiac anesthesiologist who was involved in the perioperative management of the patient and is a member of the cardiac xenotransplant research team. We discuss our views regarding important patient‐selection criteria that should be considered for future clinical trials. These criteria concern the appropriate balancing of risks and benefits with respect to patients and society, the role of an a priori psychosocial determination that allegedly predicts compliance to posttransplant medical requirements (such as medications and follow‐up appointments), and the requirements for and the proper assessment of informed and voluntary consent from critically ill patients who are presented with the prospect of life‐saving xenotransplantation when no other options are available except for hospice care. Such a Hobson's choice was presented to the recent pig‐human transplant patient. The ethics framework we propose incorporates guiding principles developed by the Ethics Committee of the International Xenotransplantation Association^7^ and builds on earlier frameworks developed for the use of unproven clinical interventions outside of clinical trials during public health emergencies.^8^ The framework also includes values regarding transparency and accountability that serve to enhance the public's trust in the scientific community, transplant investigators, and the science of transplant itself. (See table 1.)

**Table 1:** Ethical Framework to Guide Selection of Patients for Xenotransplantation Clinical Trials

*Ethical principles*	*Descriptions of the principles and related obligations*
Autonomy	Health care providers must ensure participants' understanding and appreciation of disclosed information and mitigate influences that might hinder voluntariness.
Duty of care	Health care providers must ensure that patients who have terminal heart failure receive optimal treatments. Patients who are ineligible for advanced heart failure therapies (such as an allotransplant or LVAD) or who have a high risk of dying while waiting on the transplant list must be given fair access to experimental therapies.
Distributive justice	Efforts must be made to avoid disproportionate access to experimental interventions for particular groups and to ensure that appropriate groups have fair access.
Reasonableness of the ratio of risks to anticipated benefits	With xenotransplantation, it is permissible for transplant recipients to assume a high level of risks, which are justified by the potential of direct benefits and the gain in knowledge for society.
Respect for persons	To protect patients, their close contacts, and the public, health care providers must monitor transplant recipients for potential infectious diseases. Health care providers must also respect the right of patients to withdraw from the clinical trial at any time.
Contribution to science	Investigators must share relevant data with the public and the scientific community.
Ethical oversight	Review and approvals should be obtained by the appropriate regulatory authority and the relevant ethics committees (such as clinical ethics committees and institutional review boards).
Reciprocity	If research participants endure higher burdens from volunteering in xenotransplant clinical trials, then the xenotransplant community has a moral duty to compensate these individuals for their contribution to science, for example, by providing adequate psychosocial support to patients and to third parties who are close to the patients.
Transparency	Processes of decision‐making are available to individual research participants, members of the scientific community, and oversight committees.
Reasonableness	Decisions are based on reasons (such as evidence and values) that all stakeholders agree are relevant.
Accountability	Mechanisms are in place to ensure that ethical decision‐making is sustained throughout the clincal trial process and that those responsible for making the decisions are answerable for those they did or did not make.

Commentators have proposed preliminary recommendations for selection criteria to enroll patients in clinical trials involving porcine xenotransplantation.^9^ In general, xenotransplantation should be limited to patients with life‐threatening diseases who do not have access to traditional allotransplantation.^10^ Accordingly, physiologic criteria that make patients eligible for xenotransplantation include high immunologic risk associated with cardiac allograft (such as significantly elevated antibodies against human leukocyte antigen that might be linked to prior mechanical circulatory system device placement), structural or physiological contraindications for LVAD implantation, complex congenital heart disease, and severe biventricular failure.^11^

In addition to these physiologic criteria, we propose the following ethical selection criteria for inclusion in xenotransplantation clinical trials: (a) The patient has a high likelihood of dying while on the transplant list due to the limited availability of organs. (b) The degree of the risks to the patient and to society are reasonable considering the potential direct benefits to the patient and the knowledge to be gained by society, respectively. (c) Even if the patient has been excluded from having an allotransplant based on failing to meet psychosocial criteria (which preserves the health care system's duty to steward scarce resources), the patient can be enrolled but must meet a minimal threshold for likelihood of posttransplant compliance based on a reevaluation of their psychosocial‐support resources, including those provided by the transplant research team and the medical institution. (d) The patient is assessed by qualified clinicians as capable of providing informed and voluntary consent. We turn now to an explication of these ethical criteria.

## A High Likelihood of Dying while Waiting for a Transplant

Despite an expanded donor pool, the number of patients on the waiting list for transplants has risen because of improved medical management and technological advances in bridge‐to‐therapy strategies such as LVADs and extracorporeal membrane oxygenation (ECMO), which keep patients who would otherwise die alive much longer than was once possible.^12^ A recent study showed that the one‐year survival on the heart transplant waiting list has improved during the last two decades. For example, for the period of 2011 to 2017, compared with 1987 to 1990, the one‐year survival consistently improved from 34.1 percent to 67.8 percent. Similarly, the waiting‐list outcomes for candidates with or without VADs significantly increased from 13.1 percent for 2001 to 2005 to 29.3 percent for 2011 to 2017.^13^

Despite the improvement in wait‐list survival, the risk of dying while waiting for an available organ is not trivial. We contend that, after data regarding safety and survival are obtained during the initial stages of xenotransplant clinical trials, there will be a crossover point at which it would be ethical to allow patients to decide whether to receive an experimental intervention or remain on the transplant list. To avoid an interruption between phase I and phase II of a study due to changes in study design, transplant investigators can initially adopt an adaptive trial design that incorporates plans to modify the trial eligibility criteria based on an analysis of the accumulated safety data obtained from previous subjects in the study.^14^ Accordingly, there will be stricter criteria for patients on the transplant waiting list at the initial stages of xenotransplant clinical trials, but if favorable evidence of safety and benefit emerges, then the plan will allow the criteria to be relaxed, as long as appropriate informed consent is obtained.^15^

## Benefit‐to‐Risk Analysis and Clinical Equipoise

Research ethics guidelines condition enrollment in clinical trials on a “favorable” benefit‐to‐risk analysis. However, the term “favorable” resists consistent interpretation by institutional review boards (IRBs). Talk of “balancing” and “weighing” benefits and risks is similarly ambiguous; such language gives the mistaken impression that commensurable “weights” can be assigned to these two different considerations and an unambiguous comparison of them can be made. This task is impossible. Different IRBs will assign different “weights” to risks and benefits and come to contrasting decisions about the ethical acceptability of the same research study.^16^ The ambiguity of risk assessment leads to uncertainty. For example, one study of IRB members demonstrated that, as the risks of a study increased, members’ confidence in their assessment decreased.^17^

To clarify these assessments, commentators emphasize that clinical equipoise should exist for patients undergoing clinical trials, including cardiac xenografting.^18^ Clinical equipoise requires that the benefit‐to‐risk ratio of the experimental procedure should be as “favorable” as that of the standard of care outside of the clinical trial, which for xenotransplantation clinical trials would be either heart allotransplantation or LVAD.^19^ We argue, however, that clinical equipoise will not exist for early xenotransplantation clinical trials, as the potential benefits are less and the risks are more significant than with allotransplantation or other proven advanced heart therapies. Although results from pig‐to‐baboon preclinical studies have demonstrated that the duration of survival and the performance of the heart xenograft have improved as methods to decrease rejection have improved, these results do not easily translate to humans. For humans, the benefits from xenotransplantation are probably less than those from heart allotransplantation.

And on the other side of the ledger, there are additional risks in xenotransplants, compared with allotransplants, that are largely unknown in advance of the initial attempts with xenotransplantation. For example, preclinical data cannot accurately predict the risks to patients who receive experimental porcine heart transplants and novel immunosuppressive treatment regimens. Finally, there are concerns about the transmission of pathogenic porcine viruses, which also increases risks to patients undergoing xenotransplants.

If the benefits are probably lower and the risks greater, then the benefit‐to‐risk ratio is worse for xenotransplantation than for allotransplantation. As a result, it is difficult to argue that clinical equipoise will exist in the initial xenotransplant clinical trials. Clinical equipoise might exist in phase III trials, after safety and efficacy data are obtained from the early clinical trial phases, but it is of limited use as a guide for risk‐benefit assessment in phase I and II trials.

However, more importantly, clinical equipoise is neither necessary nor sufficient for the ethics of clinical trials.^20^ Clinical equipoise is about whether the experimental intervention is equivalent to or can replace the existing standard of care.^21^ But in some types of research (such as phase I oncology trials), the benefit‐to‐risk ratio need not be “favorable” compared to the existing standard‐of‐care therapies,^22^ and the ethics of xenotransplantation clinical trials can be understood based on this model.

The above analysis, revealing that the risks are not offset by the potential benefits to patients receiving xenotransplants, raises the question whether the remaining excess risks or “net” risks that are not compensated by direct benefits to research participants can be justified by the value of the knowledge to society.^23^ As Franklin Miller and Steven Joffe have put it, “[I]s there a maximum level of net risks to consenting research subjects that can be justified by the potential social benefits from a particular scientific investigation?”^24^

We believe that an analogy can be drawn to the practice of allowing consenting adults to participate in high‐risk research during public health emergencies, as with vaccine challenge studies involving healthy human volunteers during a pandemic. High‐risk research involving xenotransplantation should be allowed because the net risks of initial xenotransplant clinical trials can be justified by the magnitude of the future benefits to society, as long as the decision‐making process is protected, informed consent is genuine, and the other relevant ethical requirements of research are present.^25^ One might object that the decision‐making capacity for giving informed, voluntary consent is better preserved in healthy volunteers participating in public health emergency studies than in patients during a terminal illness, but the potential benefits for patients entering xenotransplantation trials that offer life‐saving treatments are more significant than those for healthy volunteers in a public health emergency study. As we will argue below, the prospects for enhanced patient welfare in xenotransplant trials permit a lower threshold for the extent of certainty attached to a patient's decision‐making capacity. 

## Psychosocial Evaluation and Support

Some studies suggest that preoperative noncompliance is a useful indicator of postoperative noncompliance.^26^ Due to the importance of compliance in the posttransplant period, a psychosocial evaluation is considered an essential component of the multifaceted assessment process to determine a patient's candidacy for heart allotransplantation. The psychosocial criteria used for such an evaluation include the absence of active uncontrolled psychiatric disease, satisfactory prior compliance with medications and medical appointments, and adequate social support from a caregiver. The use of these subjective psychosocial parameters to determine whether to add a patient to the transplant waiting listing has not been trivial; a recent survey of transplant providers showed that an estimated 10 to 22 percent of transplant candidates failed to achieve the psychosocial criteria and were therefore not listed for transplant.^27^

The importance of a psychosocial evaluation is based on the principle of utility.^28^ It is alleged that deficient posttransplant compliance is a significant risk factor for graft rejection and is responsible for up to 25 percent of patient deaths after the initial recovery period. The alleged link between psychosocial support, compliance, and posttransplant survival gains importance in light of studies showing rates of noncompliance in the range between 20 and 50 percent in the post‐heart‐transplant period.^29^

When developing selection criteria for xenotransplantation clinical trials, a fundamental question is whether patients who have been denied heart allotransplantation due to inadequate psychosocial support and a history of pretransplant non‐compliance should be ineligible for xenotransplantation in clinical trials.^30^ One perspective holds that it is morally legitimate to exclude patients who had failed to meet psychosocial criteria. Undeniably, in the context of allotransplants, the importance of ensuring that patients meet the psychosocial criteria is mainly about the responsible stewardship of scarce resources. But this moral obligation does not apply to xenotransplants, as the supply of human hearts is not affected. Instead, commentators argue that the moral importance of a failure to meet psychosocial criteria in xenotransplantation stems from other relevant moral factors. For example, they maintain, including patients with adverse psychosocial factors that lead to poor posttransplant compliance would frustrate the ability to fairly evaluate the safety and efficacy of clinical trials involving xenotransplantation, and initial poor results from xenotransplant clinical trials could undermine the public confidence in xenotransplantation.

While we do not discount the importance of compliance in the post‐xenotransplant period, and although it might seem counterintuitive to enroll patients in xenotransplantation trials if they did not qualify for a heart allotransplant because of concerns about compliance, several considerations lead us to reject the use for xenotransplantation trials of psychosocial‐support parameters that were established for the workup of potential allotransplant patients. These considerations include the limited evidence that such criteria indisputably predict posttransplant compliance,^31^ significant practice variations in the use of such criteria among transplant centers,^32^ and concerns about distributive justice, as the use of psychosocial criteria might disproportionately impact the listing of patients from lower socioeconomic groups, leading to inequities.^33^

Regarding the evidence base, the assessment by psychosocial criteria relies on questionable data and is inherently subjective.^34^ It has not been possible to predict compliance and noncompliance in a fully reliable way. Although studies have purported to show that certain preoperative factors may predict posttransplant noncompliance with a high degree of probability,^35^ several recent meta‐analyses showed only a weak and inconsistent effect of social support on patient survival and graft function, and they failed to demonstrate a relationship between social support and posttransplant compliance.^36^

Second, the lack of clear guidelines for defining and evaluating social support might contribute to inconsistent and subjective processes and variation in practice among transplant centers.^37^ In the United States, there are sizable differences in rates of excluding patients from the waiting list between the different United Network for Organ Sharing regions (from 7.6 percent to 12.2 percent) and by center (21.7 percent among the top quartile of centers, compared with the average of 9.6% for all centers).^38^ Such variation is not unexpected, as there is no consensus‐based set of recommendations for determining either which psychosocial domains should be assessed during the evaluation or the set of processes and procedures to be used to conduct the evaluation. Heart transplantation programs thus vary in the range of psychosocial domains examined, the breadth of elements considered within each domain, and the processes used to implement evaluation recommendations.^39^

Finally, there is no consensus on the use of various types of formal assessments to evaluate social support. One recent survey showed that only 30 percent of respondents used a formal, validated tool to assess social support. The Stanford Integrated Psychosocial Assessment for Transplantation was used by 17 percent of respondents, while the other tools were used by fewer than 3 percent of respondents.^40^

The use of subjective psychosocial‐support requirements to determine access to xenotransplantation also raises distributive justice concerns.^41^ Applying these requirements might disproportionately impede access to the transplant list for individuals from lower socioeconomic groups, leading to inequities in care and treatment options offered to patients. For example, people from underserved populations might have a harder time demonstrating adequate social support, given caregivers’ inability to help patients come to appointments, the inflexibility of caregivers’ work schedules, and transportation costs. Additionally, people from lower socioeconomic groups might have fewer financial resources to draw on to support home‐based assistance. The various definitions of social‐support criteria might also disadvantage individuals from underserved populations, potentially impeding patients’ access to the transplant waiting list. For example, some definitions require multiple caregivers to be available for weeks to months and twenty‐four hours daily, representing a potential burden for many patients.

The application of subjective criteria might also be shaped by the implicit biases that inherently exist in medicine, leading to criteria being inconsistently applied to marginalized populations and further exacerbating disparities in access to the transplant list.^42^ These reasons have led several countries, including Canada and the European Union, to eliminate social‐support parameters from kidney transplant eligibility criteria.^43^ In a study of transplant providers, 21 percent either disagreed or were unsure when presented with a statement asserting that the process for evaluating social support at their center was impartial. In fact, in a recent survey, many transplant providers did not consider social‐support criteria fair.^44^ Greater consistency in the psychosocial evaluation both within and across programs may promote more equity in selecting patients for xenotransplantation clinical trials.

## Patient‐Centered Approaches: Interventions to Enhance Psychosocial Support

Rather than using psychosocial‐support criteria to exclude patients from xenotransplant clinical trials, accommodating the needs of patients who lack adequate psychosocial support would better balance the ethical requirement of utility against the ethical requirements of equity and respect for persons.^45^

The ability of patients and families to change their social‐support structure might depend on whether they know that their psychosocial support was considered deficient. In a recent survey, 22 percent of transplant providers believed that patients were not always informed when inadequate social support contributed to an adverse listing decision for an allotransplant.^46^ When there is a lack of transparency regarding the decision‐making process involving psychosocial assessment, patients and their families are not given an opportunity to make changes in their existing caregiving support that could make them eligible for either an allotransplant or a xenotransplant.

For patients who undergo xenotransplants, several factors suggest that the level of psychosocial support should be higher than for those receiving allotransplants. First, due to the potential risk of the transmission of zoonotic infections, surveillance entailing continual follow‐up visits and blood testing might be needed for the rest of the patients’ lives. Second, xenotransplants might impose a more significant psychosocial burden due to the unknown psychological effects of untested immunosuppressive drugs and because the patients will have received nonhuman solid organs, which is considered abhorrent by many in society. A recent mixed‐methods study involving focus groups and a representative survey of young adults showed that most people would prefer to replace their organs with materials from their own bodies and consider nonhuman animals the least desirable option. These reactions sit within a broader context of a “wisdom of repugnance,” which concerns people's intuitive responses when societal classifications of what is considered natural or not are threatened.^47^

Other commentators have suggested that several psychosocial issues might hinder the reintegration of patients into society.^48^ For example, monitoring for the remainder of xenotransplant recipients’ lives and perhaps even the patients’ temporary isolation might engender depression and other psychological reactions. Additionally, the possible risk of zoonotic infections spreading from a recipient to other community members might lead to public controversy and even fear, which may adversely impact the emotional and psychological state of people who have received xenotransplants.

The additional resources needed to ensure posttransplant compliance, including continued surveillance and management of potential psychological issues in the posttransplant period, will probably require setting the bar for demonstrating the psychosocial‐support criteria for xenotransplants at a higher level than for allotransplants. However, xenotransplant researchers and their institutions have a moral duty grounded in the principle of reciprocity to help patients and families meet this stricter bar. If research participants endure higher burdens from volunteering in xenotransplant clinical trials, then the xenotransplant community is obligated to compensate these individuals for their contribution to science. For the patient who underwent the first pig‐human heart xenotransplant, the transplant team and our medical institution made plans to provide this additional support based on this reasoning.

We offer the following recommendations regarding the use of psychosocial criteria for determining eligibility:

(a) Further studies should show how and which psychosocial‐support criteria are modifiable.
(b) The transplant selection committees should revise and standardize the assessment of the psychosocial criteria to ensure nationwide consistency. The literature offers several heuristic tools to guide evaluation.^49^ Although such tools can enhance consistency across different transplant programs, the evidence linking these tools to enhanced compliance or improved posttransplant outcomes is weak. More research is needed to support their widespread use.^50^
(c) Medical institutions should engage the public in developing fair psychosocial criteria and make transparent the processes involved in their use.
(d) The transplant team should involve its hospital's clinical ethics committee in using subjective psychosocial‐support criteria in the decision to list a patient for xenotransplantation.
(e) Xenotransplant clinical trial research teams should allocate a portion of their budgets to the provision of psychosocial support to patients undergoing xenotransplantation.

## Informed and Voluntary Consent in Xenotransplantation

A patient can make informed decisions voluntarily when they have an adequate understanding and appreciation of a situation and possess the cognitive abilities to weigh trade‐offs. The context of xenotransplantation poses several challenges to voluntary decision‐making. These include the overwhelming amount of information a patient must process coupled with a compromised decision‐making capacity caused by an interrupted sleep‐wake cycle and being in the hospital for an extended period (which together can produce delirium). The challenge will be even greater for patients who are being administered intravenous sedative medications or are receiving dialysis or ECMO. “Adequate” understanding will be harder yet to achieve from an informed consent process that requires navigating a lengthy text or discussion. The sheer uncertainty of the risks of posttransplant zoonotic infections and novel immunosuppressive drugs can present a formidable challenge to comprehension. And patients also need to be made aware that in case of severe and unknown infections, they could be required to quarantine, fully isolated from social life, keeping physically distant from even their immediate family members and intimate friends, and unable to move forward with some important personal goals, like pregnancy, marriage, and school graduation.

However, these threats to understanding do not necessarily mean that patients cannot give informed consent. If decision‐making capacity is specific to a decision and measured on a sliding scale, whereby less‐reasonable decisions that adversely affect patient welfare require greater certainty of a patient's decision‐making capacity, then the decision to accept xenotransplantation when no other options are available does not require a high threshold of certainty, as the surgery can promote patient welfare.^51^

Voluntariness might also be impeded by substantial controlling influences on a patient, including physiologic conditions like terminal heart failure, situational factors such as the desperation induced by an absence of other life‐saving options, and other people involved in the decision‐making process, such as families and clinicians.^52^ Insofar as patients considering xenotransplantation are in a desperate situation that might affect voluntariness,^53^ they are similar to oncology patients who are offered promising “treatments” in a research setting. Not all influences, however, are necessarily controlling. For example, influences from other persons might amount merely to persuasion, which is ethically appropriate compared with coercion or manipulation of information.^54^ Additionally, family influences can be considered morally legitimate based on a concept of relational autonomy.^55^

## Therapeutic Misconception

It is widely accepted that informed consent to participate in research requires understanding the differences between research and standard clinical care.^56^ Paul Appelbaum and Charles Lidz first described the therapeutic misconception as the mistaken belief “that the research, like the therapy [patients] have received previously, is designed and will be executed in a manner of direct benefit to them”^57^ rather than with the primary goal of producing knowledge. Empirical studies have documented the presence of therapeutic misconception among severely ill patients entering clinical trials.^58^ A typical example of the therapeutic misconception occurs when patients with end‐stage illnesses who have exhausted all standard treatments harbor unreasonable expectations of medical benefit from experimental clinical trials. Such a situation might indicate a breakdown of informed consent stemming from investigators’ failure to explain that direct medical benefit from a trial is not the primary goal and might be unlikely to achieve.

Gail Henderson and colleagues have identified five dimensions of research that individuals should understand prior to enrolling in a clinical trial.^59^ These include scientific purpose, study procedures, uncertainty of risks, adherence to the protocol, and the clinician's role as an investigator.

A further issue regarding informed consent in xenotransplantation clinical trials involves the right of participants to withdraw from the trial at any time. The problem is that research participants will need to undergo surveillance for the rest of their lives to check whether they harbor virulent porcine viruses that could be transmitted to others. They will, in effect, be in a clinical trial for the rest of their lives. To enhance the protection of society from viral infections, there might be an inclination to condition enrollment in xenotransplantation clinical trials on a patient's promise of fidelity to remain in the research. However, a recognized ethical requirement of clinical research is that research participants should not be denied their right to withdraw from the study at any time.

A final issue unique to xenotransplantation is whether documented informed consent is required from patients’ close contacts, given their possible exposure to zoonotic viral infections and the requirement that they undergo surveillance monitoring, thereby compromising their right to privacy. We believe that instead of consent (a legal agreement), only assent, indicating their agreement or willingness to be part of the clinical study, is required from these third parties. The rights of patients to participate in xenotransplantation should not be conditioned on the consent of third parties. In contrast, hospital staff should be required to provide documented informed consent that they accept the risks of caring for xenotransplantation patients, and the medical institution should offer reasonable accommodations to those who refuse to care for these patients.

We offer the following recommendations for procuring informed and voluntary consent:

(a) Assessments should be made regarding the patients’ knowledge, understanding, capacity, and voluntariness to engage in decision‐making about transplantation. For example, after a potential xenotransplant recipient is provided information about a xenotransplantation clinical trial, their understanding of the information should be assessed by having them repeat it in their own words.
(b) The informed consent process should occur over several days to ensure that patients have enough time to process the information about the purpose of the clinical trial, the experimental study procedures, the potential risks and potential benefits, and the possible need for surveillance for viral infections for the rest of their life.
(c) A comprehensive psychiatric determination should be made of the patient's decision‐making capacity to accept enrollment in the clinical trial.
(d) The transplant team should make clear to patients that, while a pig‐heart xenotransplant could be beneficial and prolong life, it is an experimental procedure rather than standard clinical care; that one could die from an immediate or delayed rejection of the heart; that the patient's quality of life might be worsened; and that the patient will need to take immunosuppressants, which pose an additional set of risks. Patients must also be informed that they will receive experimental antirejection drugs that suppress the immune system in ways different from how other immunosuppressants work and that there is limited information on the safety and effectiveness of these newer immunosuppressants. Finally, patients should be aware of risks of prolonged, untreatable infections from unique pathogens and that there may be unknown risks from xenotransplantation.
(e) Patients need to appreciate that, while transplantation has been deemed to perform well in preclinical studies involving nonhuman primates, those results might not be transferable to the human situation.
(f) Patients should realize that an alternative to undergoing xenotransplantation includes receiving medications that optimize comfort at the end of life if the patient chooses not to undergo xenotransplantation.
(g) If the clinical investigator is the patient's treating physician, the investigator must be conscientious about presenting a balanced perspective on the options available to the patient. Another option would be to include in the consenting process a second qualified professional who is not involved in the clinical trial to assist in presenting the options available to the patient and to minimize any possibility of coercion, even if unintended.
(h) A representative of the hospital's clinical ethics committee should be involved in the informed consent process.

## A Unique but Not Impossible Ethical Challenge

The ethical issues with participation in high‐risk research involving xenotransplantation are unique and require careful deliberation among the transplant investigators, psychosocial providers, psychiatrists, and ethicists. To facilitate that deliberation and ensure that the wide range of ethical issues are given due consideration, we have proposed a framework that incorporates both substantive and procedural ethical values and can be used to address patient selection criteria for xenotransplant clinical trials.

The assessment of benefits and risks should ensure that a trial has significant social benefit to justify excess risks that are not justified by a compensatory potential benefit to patients. The transplant team should have a heightened sensitivity to the distributive and procedural justice concerns of psychosocial criteria used in transplant listing decisions. Efforts should be made to enhance the social support available to a patient, rather than using psychosocial criteria solely to determine the listing of potential candidates. Procuring informed and voluntary consent should represent a deliberative process that occurs over several days, with participation by a representative of the hospital's clinical ethics committee. Discussions of these ethical issues among ethicists and transplant investigators in combination with further technological advances in xenotransplantation can move the status of xenotransplantation forward to a reality.
